# Anemia, costs and mortality in Chronic Obstructive Pulmonary Disease

**DOI:** 10.1186/1478-7547-4-17

**Published:** 2006-10-16

**Authors:** Michael T Halpern, Marya D Zilberberg, Jordana K Schmier, Edmund C Lau, Andrew F Shorr

**Affiliations:** 1 Exponenet, 1800 Diagonal Road, Suite 300, Alexandria, VA 22314, USA; 2 Ortho Biotech 430 Rt. 22 East Bridgewater, NJ 08807-0914, USA; 3 Exponent, 149 Commonwealth Dr., Menlo Park, CA 94025, USA; 4 Pulmonary and Critical Care Medicine, Room 2A-38D, Washington Hospital Center, 110 Irving St., NW, Washington, DC 20010, USA

## Abstract

**Background:**

Little is known about cost implications of anemia and its association with mortality in chronic obstructive pulmonary disease (COPD). This claims analysis addresses these questions.

**Methods:**

Using the the US Medicare claims database (1997–2001), this study identified Medicare enrollees with an ICD-9 diagnosis of COPD. Concomitant anemia was identified based on ICD-9 codes or receipt of transfusions. Persons with anemia secondary to another disease state, a nutritional deficiency or a hereditary disease were excluded. Medicare claims and payments, resource utilization and mortality were compared between COPD patients with and without anemia.

**Results:**

Of the 132,424 enrollees with a COPD diagnosis, 21% (n = 27,932) had concomitant anemia. At baseline, anemic patients were older, had more co-morbidities and higher rates of health care resource use than non-anemic individuals with COPD. In a univariate analysis annual Medicare payments for persons with anemia were more than double for those without anemia ($1,466 vs. $649, p < 0.001), the direction maintained in all categories of payments. Adjusting for demographics, co-morbidities, and other markers of disease severity revealed that anemia was independently associated with $3,582 incremental increase per patient (95% CI: $3,299 to $3,865) in Medicare annual reimbursements. The mortality rate among COPD patients with anemia was 262 vs. 133 deaths per 1,000 person-years among those without anemia (p < 0.001).

**Conclusion:**

Anemia was present in 21% of COPD patients. Although more prevalent in more severely ill COPD patients, anemia significantly and independently contributes to the costs of care for COPD and is associated with increased mortality.

## Background

Chronic obstructive pulmonary disease (COPD) is a major cause of both morbidity and mortality. In the United States, more than 10 million adults suffer from COPD and it is the fourth most common cause of death [1]. Because of the continued high rate of smoking coupled with the aging of the population, analysts predict that COPD will account for an increased proportion of deaths in the near future [2]. With respect to morbidity, COPD impairs both functional status and quality of life [1]. Economically, the costs of COPD associated with medical care and lost productivity are staggering, exceeding $37 billion annually in the United States [3].

Anemia is commonly present among individuals with chronic illnesses. In a broad range of medical conditions, concomitant anemia is increasingly being recognized as a risk factor associated with increased mortality [4,5]. There is also growing evidence of increased health care utilization and costs associated with anemia in disease states such as chronic kidney disease, HIV, rheumatoid arthritis, inflammatory bowel disease, congestive heart failure, and cancer [6-8]. However, less is known regarding the impact of anemia on mortality, morbidity, and costs of care for patients with COPD. Cote and colleagues observed that anemia was associated not only with increased mortality but also with worsened dyspnea scores and functional capacity as measured by the 6-minute walking distance in a COPD cohort [9,10]. The effect of anemia was independent of age, forced expiratory volume in one second, and functional status. Similarly, a review of a French national database indicated that subjects with severe oxygen-dependent COPD and anemia experienced an increased risk of hospitalization and short-term mortality compared to those without anemia [11].

Despite the growing awareness of an association between worse clinical and functional outcomes and anemia in COPD patients, the financial implications of anemia in COPD have not been examined previously. To determine what incremental costs, if any, were associated with anemia in COPD patients, and to confirm the association of anemia with clinical and functional outcomes in a larger population, data from Medicare beneficiaries in the United States were analyzed.

## Methods

### Overview and database description

Medicare claims data for persons with COPD were analyzed; costs and outcomes for patients with anemia were compared to those of patients without anemia. The data source was the Medicare 5% beneficiary encrypted files (BEF) from a recent five-year period (1997–2001), available from the Centers for Medicare and Medicaid Services (CMS). The BEF represents a systematic 5% sample of all Medicare enrollees, which corresponds to essentially all U.S. residents age 65 and older as well as select groups younger than age 65 (e.g., patients with end-stage renal disease or certain disabilities). This analysis included all categories of the BEF data: Durable Medical Equipment (DME), Inpatient Facility, Hospice, Outpatient Facility, Home Health Agency (HHA), Skilled Nursing Facility (SNF, nursing home), and Physician/Supplier (Part B) claims.

### Subjects and definitions

To be included in the cohort, beneficiaries had to have at least two claims with diagnoses (primary or secondary) of COPD from either Part B (physician/supplier) or outpatient files. Persons under 65 years of age were excluded in order to limit the impact of age as a source of heterogeneity in the study population. Those persons who were not residents of either one of the 50 states or the District of Columbia were also excluded. In addition, those with known nutritional or hereditary anemias or with other disease states that are commonly associated with anemia (end stage renal disease, cancer, and gastrointestinal bleeding) were eliminated. COPD patients with co-morbid anemia were identified in two ways: claims with ICD-9 codes specific for anemia, or receipt of blood transfusions not associated with other apparent causes (e.g., acute blood loss from surgery, trauma, etc.). The appendix lists the specific ICD-9 codes used to identify and define the study cohort.

**Figure 1:**
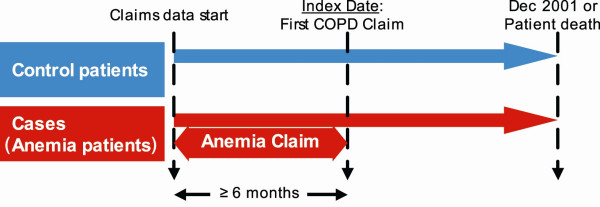
Anemia Among COPD Patients: Case and Control Identification.

To control for variation in the utilization of medical care services over time and the inherent inter-patient variability in the need for healthcare encounters, an "index date" was determined for each patient. For those with COPD but without anemia, the date of the first medical claim with a COPD diagnosis at least six months after entering the database (at the time of either Medicare enrollment or the start of the data set's availability) served as the index date. For their anemic counterparts, the index date was the first medical claim with a COPD diagnosis that was both six months after that patient entered the data set and after or concurrent with the claim for anemia. Only patients with at least six months of Medicare enrollment prior to their index date and at least 12 months of enrollment following their index date were included in the analysis. Details regarding selection of case and control patients are illustrated in Figure 1.

### Outcome measures

Costs to the Medicare system for all medical care served as the primary endpoint. To determine the attributable effect of anemia, costs among persons with anemia and COPD were compared to costs among non-anemic COPD patients. Costs of care were stratified by the type of service involved (e.g., durable medical equipment, inpatient facility, hospice, etc.). Costs based on claims (submitted charges) and actual payments (reimbursements) were analyzed separately. All costs were inflated to 2004 U.S. dollars by using the medical care component of the Consumer Price Index.

Rates of resource utilization represented a secondary endpoint. We evaluated the effect of anemia on the frequency of the use of selected resources, including outpatient visits, hospital admissions, ICU care, and home health claims.

The association of anemia with survival in the study group was also examined. Duration of survival was calculated from index date to time of death and based on the number of quarters of Medicare enrollment until the end of either follow-up data on December 31, 2001, termination of Medicare entitlement, or death.

### Co-variates of interest

In addition to the presence or absence of anemia, patient demographics (e.g., age, gender, race/ethnicity) and the distribution of other patient co-morbidities as categorized by organ system using ICD-9 coding wee also assessed. Although precise measures of COPD disease severity (e.g., pulmonary function test results) are not present in the Medicare claims database, surrogate measures of disease severity, including use of ventilation support, episodes of serious pulmonary respiratory infections (chronic bronchitis and pneumonia), and numbers of hospitalizations and emergency room visits for COPD in the six months prior to the index date (as defined above) were utilized to control for baseline disease severity in comparisons of COPD patients with versus without anemia.

### Statistics

Continuous data were analyzed with the Student's t-test or the Mann-Whitney U test, as appropriate. Chi-square test was used to compare categorical variables. All event rates and frequencies were expressed as events per 1,000 person-years or as annualized rates to allow for comparisons between the two groups. Multivariate linear regression modeling was used to calculate the independent costs of care associated with anemia in COPD. Regressions included demographic variables and the disease severity surrogate measures noted above and identified *a priori *as biologically important confounders that would affect resource use in COPD. To determine survival, total person-years of follow up, total deaths, and death rates (per 1,000 person-years) were calculated using the Kaplan-Meier method. For this survival analysis, surviving enrollees were censored at December 31, 2001 (the end of the data capture period) or at the end of their Medicare enrollment if before that date. Survival curves were compared with the log-rank test. P values of <0.05 were assumed to represent statistical significance, and 95% confidence intervals are reported where appropriate. All analyses were conducted using SAS version 9.1 (SAS Institute, Cary, NC).

## Results

The cohort included 132,424 patients with COPD. Approximately 21% (n = 27,932) were identified as having concomitant anemia that was not associated with nutritional or hereditary anemias, acute blood loss, end-stage renal disease, or malignancy. As shown in Table 1, anemic patients tended to be older and were more likely to be female. In the quarter prior to the index date, anemic patients were 1.71 times (95% CI: 1.63–1.79) more likely to have needed mechanical ventilation than non-anemic patients. With respect to comorbid conditions (Table 1), neurologic, respiratory, and musculoskeletal diagnoses were more frequent in those without anemia. Skin and GI conditions were similar between the two groups, and all other conditions occurred more often in the anemic population.

**Table 1:** Baseline Patient Characteristics

**Variable**	**COPD with Anemia (n = 27,932)**	**Non-anemic COPD (n = 104,492)**	**P value**
***Demographics***			
Age, mean (SD), years	77.5 ± 7.8	74.7 ± 7.6	<0.001
Age distribution (years, %)			
65–69	19.0%	31.5%	<0.001
70–74	20.1%	23.0%	
75–79	21.4%	18.7%	
80–84	18.7%	13.7%	
> 84	20.6%	12.1%	
Male (%)	34.2%	42.4%	<0.001
***Pulmonary Characteristics of Interest Present in the Six Month Baseline Period***			
Concomitant asthma diagnosis	25.2%	22.6%	<0.001
Use of supplemental oxygen	9.8%	3.7%	<0.001
***Comorbidities, % (by ICD-9 coded organ system) Present in the Six Month Baseline Period***			
Infectious and parasitic	1.7%	1.4%	<0.001
Skin disorders	2.3%	2.2%	NS
Digestive disorders	2.5%	2.5%	NS
Mental disorders	3.3%	2.9%	<0.001
Genitourinary disorders	3.9%	3.3%	<0.001
Blood diseases	4.3%	0.8%	<0.01
Nervous system	4.4%	6.3%	<0.001
Musculoskeletal disorders	7.7%	9.0%	<0.001
Other respiratory disorders	8.3%	8.8%	<0.001
Endocrine and metabolic	8.5%	7.9%	<0.001
Circulatory disorders	26.8%	26.0%	<0.01

Abbreviations: COPD – Chronic obstructive pulmonary disease, NS – not significant

Specific rates of health care utilization during the six months prior to the index date (i.e., prior to identification of these patients having anemia) were greater for patients with co-morbid anemia (Table 2). During the six-month baseline period, anemic persons had significantly more claims for hospitalizations, ICU care, and episodes of acute exacerbations of COPD (AECB) and pneumonia. For example, being classified with co-morbid anemia was associated with a greater need for ICU care (15.5 claims per 100 patients with anemia during the baseline period vs. 7.3 claims per 100 patients without anemia, p < 0.001). These differences indicate greater baseline severity of illness among patients with COPD and anemia.

**Table 2:** Baseline Resource Utilization

**Characteristic**	**COPD with Anemia (n = 27,932)**	**Non-anemic COPD (n = 104,492)**	**P value**
# of hospitalizations in 6 months before index date			
Claims per 100 patients	63.2	29.7	<0.001
Claims per 12 month period	1.26	0.59	<0.001
# of ICU encounters in 6 months before index date			
Claims per 100 patients	15.5	7.3	<0.001
Claims per 12 month period	0.31	0.15	<0.001
# of AECB medical encounters in 6 months before index date			
Claims per 100 patients	27.0	16.0	<0.001
Claims per 12 month period	0.54	0.32	<0.001
# of pneumonia medical encounters in 6 months before index date			
Claims per 100 patients	83.5	34.9	<0.001
Claims per 12 month period	1.67	0.79	<0.001

Abbreviations: AECB – acute exacerbations of chronic bronchitis, COPD – chronic obstructive pulmonary disease, ICU – intensive care unit

**Figure 2:**
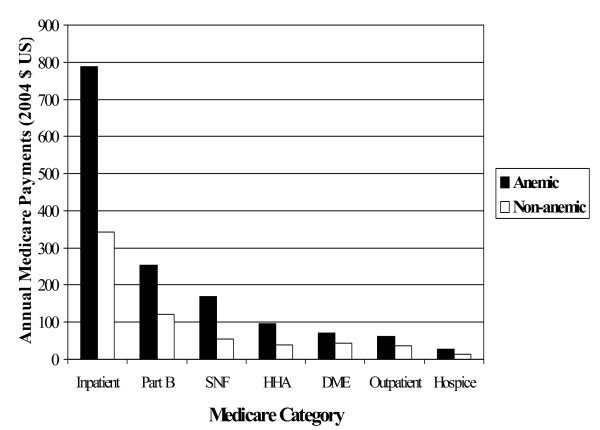
The Impact of Anemia on Annual Medicare Payments for COPD Patients by Category. Abbreviations: DME – durable medical equipment, HHA – home health assistance, SNF – skilled nursing facility.

Table 3 presents the unadjusted total claims (charges) and reimbursements (payments) after the index date (in 2004 dollars). Average annual payments from Medicare were substantially higher among those with anemia. Medicare spent $1,466 per patient per year among COPD patients with anemia vs. $649 per patient per year in non-anemic COPD patients (p < 0.001). Resource utilization rates among patients with anemia (Table 4.), as well as payments for COPD patients with anemia were higher in every resource utilization category (Figure 2). Rates of mechanical ventilation, ICU care, emergency department visits, and other resource use were also higher among COPD patients diagnosed with anemia (Table 4).

**Table 3:** Total Medicare Claims (Charges) and Reimbursements (Payments) for COPD Patients with versus without Anemia After Index Date

	**COPD with Anemia (n = 27,932)**				**Non-anemic COPD (n = 104,492)**			
Type of Resource Utilization	Total Number of Claims	Total Claim Amount	Average Claim per 12 M Enrollment	Average Payment per 12 M Enrollment	Total Number of Claims	Total Claim Amount	Average Claim per 12 M Enrollment	Average Payment per 12 M Enrollment

DME	200,952	$78,660,190	$162	$71	693,549	$262,757,934	$101	$44
HHA	34,113	$59,075,953	$121	$96	83,185	$129,983,681	$50	$39
Hospice	5,182	$13,823,214	$28	$27	13,204	$35,768,474	$14	$13
Inpatient	43,129	$965,219,860	$1,985	$788	107,793	$2,061,627,072	$789	$342
Outpatient	181,577	$110,476,975	$227	$62	700,001	$375,269,221	$144	$36
Part B	1,347,897	$324,691,886	$668	$254	3,781,373	$878,749,293	$336	$121
SNF	18,907	$153,345,595	$315	$168	34,515	$280,467,242	$107	$54
Total*	1,831,757	$1,705,293,673	$3,506	$1,466	5,413,620	$4,024,622,917	$1,541	$649

*Claims and payments significantly different between patients with and without anemia, p < 0.001 Note: 1997–2001 data inflation-adjusted to 2004 $ using the Medical Consumer Price Index

Abbreviations: 12 M – 12 months of continuous Medicare enrollment, DME – durable medical equipment, HHA – home health assistance, SNF – skilled nursing facility

**Table 4:** Resource Utilization After Index Date

	**COPD with Anemia (n = 27,932)**		**Non-anemic COPD (n = 104,492)**		**P value**
	
Type of Resource Utilization	Total Number*	Annual Rate	Total Number*	Annual Rate	
Outpatient Visits	151,930	3.76	678,416	3.12	<0.001
Emergency Department Visits	19,878	0.49	74,951	0.34	0.038
Hospitalizations	36,970	0.91	106,230	0.49	0.036
ICU Admissions	9,062	0.22	25,469	0.12	<0.001
Ventilation Episodes	2,270	0.06	6,188	0.03	<0.001
Home Health Care Visits	1,374	0.03	4,302	0.02	<0.001
Nursing Home Admissions	4,342	0.11	10,298	0.05	<0.001
Medication Prescriptions (non-self-administered)	26,689	0.66	89,361	0.41	0.334
Transfusions	6,348	0.16	6,394	0.03	<0.001

*From Index Date to end of data, end of 12 month study period, or end of Medicare enrollment

Abbreviations: COPD – Chronic obstructive pulmonary disease, ICU – intensive care unit

**Figure 3:**
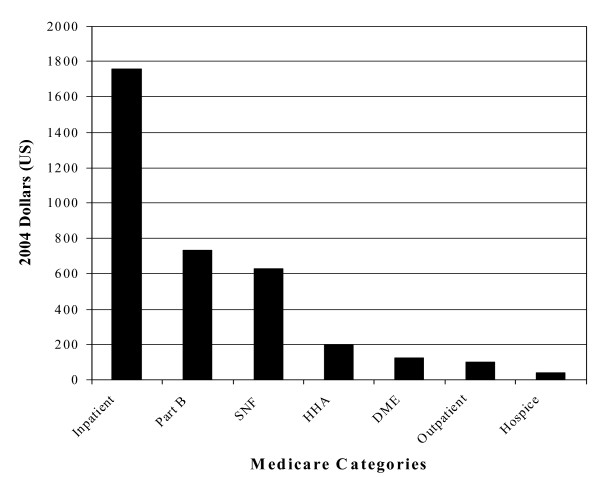
Adjusted Incremental Difference in Medicare Payments for COPD by Category. Abbreviations: DME – durable medical equipment, HHA – home health assistance, SNF – skilled nursing facility.

Costs for Medicare enrollees in both groups, with and without anemia, rose after the index date. The increase in claims was 78.1% for COPD patients with anemia and 50.9% for patients without anemia (p < 0.001); the increase in payments was 71.5% for patients with anemia and 48.5% among patients without anemia (p < 0.001). Using linear regression to control for baseline differences in severity of illness (e.g., number of co-morbid illnesses, need for ventilation support, and episodes of pneumonia and acute exacerbations of chronic bronchitis in the six months prior to the index date) and preceding health care use (e.g., frequency of hospital admissions and emergency room care for COPD) the incremental independent annual per patient charges and reimbursements associated with anemia among COPD patients were calculated to be $8,811 (p < 0.001, 95% CI: $8,136 to $9,487) for charges and $3,582 (p < 0.001, 95% CI: $3,299 to $3,865) for payments. The independent contribution of anemia to costs in COPD was present in all categories of resource utilization (Figure 3). Of note, anemia care (defined as medical claims with an anemia diagnosis) did not appear to account directly for these incremental differences in cost. Less than 1% of the over 890,000 claims for the co-morbid anemia population filed in the 12 months after the index date had a diagnosis code specific for anemia.

Persons with anemia complicating their COPD had a nearly two-fold increase in death rates (262 per 1,000 person-years of follow-up) compared to the non-anemic individuals (133 deaths per 1,000 person-years of follow-up, p < 0.001). The association of anemia and mortality was stronger among younger Medicare patients; among those 65–69 years of age, anemia was associated with an odds ratio for death of 2.07 (95% CI: 1.94 to 2.22). In contrast, among the 80–84 year old cohort the odds ratio for mortality in those with anemia and COPD was 1.42 (95% CI: 1.36 to 1.49).

## Discussion

This analysis of the Medicare population indicates that anemia is frequent in COPD, occurring in 21% of the current study patients. More importantly, the presence of anemia is associated with substantial economic burden and increased resource utilization among these beneficiaries. Although persons with COPD and anemia are more severely ill than their non-anemic counterparts, the association of anemia and costs is independent of multiple important potential confounders. Moreover, the majority of incremental costs among anemic COPD patients is not coded as direct care for anemia. In addition, co-morbid anemia is associated with increased mortality for COPD patients.

Prior research into anemia in COPD is limited. John and colleagues examined a population of 101 COPD patients and estimated the prevalence of anemia at 13% [12]. They noted that neither age nor spirometric measures differed between anemic and non-anemic persons. Those with lower hemoglobins, however, had more extensive systemic inflammation as measured by both IL-6 and C-reactive protein levels [12]. The variability in the prevalence of anemia between theirs and the current study likely reflects differences in definitions. John et al. relied on actual hemoglobin measurements while the current administrative data are limited to ICD-9 codes. Additionally, the mean age in their analysis was approximately 61 years; the present study's cohort was older since it derived from a Medicare sample, and the prevalence of anemia is known to increase with increasing age [13]. Somewhat contrary to the findings by John, a recent study by Chambellan and coworkers in a cohort of COPD patients with severe oxygen-dependent COPD demonstrated that anemia (defined by laboratory values according to the WHO criteria [14]), present in 8.2–12.6%, was in fact correlated with age and disease severity in this population, and, consistent with the current findings, hematocrit was an independent predictor of survival, hospital admission rate and duration of hospitalization [11]. Cote and co-workers, focusing on a population from a US Veterans Affairs pulmonary clinic, estimated the prevalence of anemia at 30% [9]. As in the John et al. study, these investigators had patient level hemoglobin data available. Similar to the present findings, Cote et al. reported that anemic COPD patients had more co-morbid illnesses [9,10]. They also concluded that the presence of anemia independently correlated with mortality [9,10].

While it is likely that CKD, advanced age or certain medication exposure may play a role in the development of anemia in this population, at least one study points to the possible role of inflammation and resistance to elevated levels of erythropoietin [12]. Etiology notwithstanding, our study suggests anemia is highly prevalent in the COPD population and associated with worse clinical and economic outcomes.

The present study builds on prior data and suggests that anemia is common in COPD. As it was necessary in the present study to use ICD-9 codes rather than hemoglobin values, these findings likely underestimate the frequency of anemia. Examining a very large sample, however, adds to the robustness of the conclusions. Furthermore, this study was able to control for many confounders that the earlier reports could not assess. Specifically, the current analysis accounted for prior health care utilization and factors associated with COPD severity, such as the frequency of episodes of AECB and pneumonia. This study is also unique in that it measures the economic impact of anemia in COPD and describes the increased utilization of various healthcare resources among patients with anemia. Finally, this study was also able to corroborate previous work indicating that COPD patients with anemia are at higher risk for death.

The association between anemia and increased risk of mortality seen here among COPD patients parallels previous reports of links between anemia and decreased survival in other disease states. For example, in subjects with left ventricular dysfunction, anemia independently adversely affects survival [15,16]. Similarly, in chronic kidney disease, anemia not only affects quality of life but also worsens survival [17]. This study's finding of relatively high costs of medical care for individuals with COPD (even those who do not have anemia) is not surprising, given that both increased age and decreased health status are associate with increased costs in several different components of healthcare [18]. Specifically, in COPD, direct healthcare costs in the United States were estimated to exceed $18 billion in 2002 and COPD led to nearly 750,000 hospital admissions [19]. These hospitalizations, along with treatment of AECB, are major drivers of costs. Results of this study, indicating that inpatient care was the single largest category of Medicare claims for this population, support findings from previous studies.

This study has several important limitations. First, as a retrospective study, it is exposed to various forms of bias. Second, since the data source was administrative claims data, findings may have been affected by coding bias; that is, only those COPD patients and the subset with anemia who had Medicare claims with appropriate ICD-9 diagnosis codes could be identified, though the presence of such codes could not guarantee the appropriateness of these diagnoses. The large sample size, however, balances these concerns, and for some endpoints, such as mortality and hospitalizations, the results are in accordance with other studies. Further, prior large chart audits indicate that ICD-9 codes for COPD are actually both specific and sensitive for this condition [20]. Third, as mentioned earlier, anemia was defined based on ICD-9 coding. As a majority of US pulmonologists participating in a recent survey did not classify anemia as being present among COPD patients using a threshold hemoglobin value of 12 g/dL, it is unlikely that this study overestimated the true prevalence of anemia in this population and may well have underestimated it [21]. If clinicians do not believe anemia is present, they are unlikely to use an anemia diagnosis code for the provided services. Additionally, there is no reason to believe, a priori, that bias in recording anemia would lead to the more "expensive" patients consistently being labeled as anemic. Finally, no specific measures of disease severity were available. Hence surrogate markers of severity of illness, which correlate with outcomes and have been validated in previous health services research in COPD, were utilized. Moreover, other than the recently proposed BODE index, no global, validated severity score in COPD exists [22]. Despite these best efforts at adjusting for identifiable confounders, residual confounding cannot be excluded as a potential source of the independent relationship between anemia and worsened outcomes shown in this study.

In conclusion, anemia is a comorbidity commonly associated with COPD. Although the nature of this retrospective observational study precludes any inference of causality, it is clear that anemia in this patient population is associated with increased healthcare costs and resource utilization, as well as worsened survival. Future research is warranted to define more accurately the epidemiology and the etiology of anemia in COPD, to evaluate the impact of anemia on quality of life among persons with COPD, and to explore options for and effects of treating anemia in COPD.

## Appendix

Codes used to identify study population

• COPD Patients: ICD9 491–492, 496

• Presence of Anemia: ICD9 280–281, 283–285, or blood transfusion w/o major surgery or trauma (blood pint>0 but DRG # 1–8, 27–30, 36–42, 49–63, 72, 75–77,83–84, 104–120, 146–171, 191–201, 209–236, 257–270, 280–282, 285–293, 302–315, 334–345, 353–365, 370–394, 400–410, 413–418, 424, 439–455, 468–511)

Exclude ESRD (Medicare Status Code = 10 aged w/o ESRD or ICD9 996.68, 996.73, V45.1, V56), malignancies (ICD9 140–239), HIV (ICD9 042, 079.53), GI bleeding (ICD9 456.0, 456.20, 530.21, 530.7)

## Competing interests

This project was funded by a grant from Ortho Biotech Clinical Affairs, LLC, Bridgewater, NJ. Dr. Shorr has served as a consultant to Ortho Biotech. Dr. Zilberberg is an employee of Ortho Biotech and a stockholder of Johnson & Johnson, its parent company.

## Authors' contributions

MH contributed to the design of the study, executed the principle analyses, and facilitated analysis and manuscript preparation. MZ conceived of the study, helped design the analytic plan, and secured funding. JS assisted in data analysis and interpretation and helped to draft the manuscript. EL provided statistical expertise and contributed statistical methods and data interpretation. AS helped with study design, data analyses, clinical interpretation, and manuscript preparation.

## References

[B1] Pauwels RA, Rabe KF (2004). Burden and clinical features of chronic obstructive pulmonary disease (COPD). Lancet.

[B2] Pauwels RA, Buist AS, Calverley PM, Jenkins CR, Hurd SS (2001). Global strategy for the diagnosis, management, and prevention of chronic obstructive pulmonary disease. NHLBI/WHO Global Initiative for Chronic Obstructive Lung Disease (GOLD) Workshop summary. Am J Respir Crit Care Med.

[B3] National Heart Lung and Blood Institute (2004). Morbidity & Mortality: 2004 Chart Book on Cardiovascular, Lung, and Blood Diseases.

[B4] McCullough PA, Lepor NE (2005). The deadly triangle of anemia, renal insufficiency, and cardiovascular disease: implications for prognosis and treatment. Rev Cardiovasc Med.

[B5] McCullough PA, Lepor NE (2005). Piecing together the evidence on anemia: the link between chronic kidney disease and cardiovascular disease. Rev Cardiovasc Med.

[B6] Nissenson AR, Wade S, Goodnough T, Knight K, Dubois RW (2005). Economic burden of anemia in an insured population. J Manag Care Pharm.

[B7] Ershler WB, Chen K, Reyes EB, Dubois R (2005). Economic burden of patients with anemia in selected diseases. Value Health.

[B8] Lyman GH, Berndt ER, Kallich JD, Erder MH, Crown WH, Long SR, Lee H, Song X, Finkelstein SN (2005). The economic burden of anemia in cancer patients receiving chemotherapy. Value Health.

[B9] Cote C, Zilberberg M, Mody S, Celli B (2005). Anemia is a predictor of mortality in patients with COPD. Proceedings of the American Thoracic Society.

[B10] Cote C, Zilberberg M, Mody S, Celli B (2005). Anemia is associated with increased breathlessness and decreased 6-minute walk distance in COPD patients. Proceedings of the American Thoracic Society.

[B11] Chambellan A, Chailleux E, Similowski T (2005). Prognostic value of the hematocrit in patients with severe COPD receiving long-term oxygen therapy. Chest.

[B12] John M, Hoernig S, Doehner W, Okonko DD, Witt C, Anker SD (2005). Anemia and inflammation in COPD. Chest.

[B13] Yip R, Johnson C, Dallman PR (1984). Age-related changes in laboratory values used in the diagnosis of anemia and iron deficiency. Am J Clin Nutr.

[B14] World Health Organization (2001). Iron deficiency anaemia: assessment, prevention and control. A guide for programme managers. Document WHO/NHD/01.3.

[B15] Maggioni AP, Opasich C, Anand I, Barlera S, Carbonieri E, Gonzini L, Tavazzi L, Latini R, Cohn J (2005). Anemia in patients with heart failure: prevalence and prognostic role in a controlled trial and in clinical practice. J Card Fail.

[B16] Locatelli F, Pozzoni P, Tentori F, del Vecchio L (2003). Epidemiology of cardiovascular risk in patients with chronic kidney disease. Nephrol Dial Transplant.

[B17] Silverberg D (2003). Outcomes of anaemia management in renal insufficiency and cardiac disease. Nephrol Dial Transplant.

[B18] Stewart ST (2004). Do out-of-pocket health expenditures rise with age among older Americans?. Gerontologist.

[B19] Mapel D, Chen JC, George D, Halbert RJ (2004). The cost of chronic obstructive pulmonary disease and its effects on managed care. Manag Care Interface.

[B20] Wilchesky M, Tamblyn RM, Huang A (2004). Validation of diagnostic codes within medical services claims. J Clin Epidemiol.

[B21] Zilberberg M, Pisano M (2005). Pulmonologists' perceptions of anemia in COPD: a survey. Value Health.

[B22] Celli BR, Cote CG, Marin JM, Casanova C, Montes de Oca M, Mendez RA, Pinto Plata V, Cabral HJ (2004). The body-mass index, airflow obstruction, dyspnea, and exercise capacity index in chronic obstructive pulmonary disease. N Engl J Med.

